# The Strength and Fire Properties of Paper Sheets Made of Phosphorylated Cellulose Fibers

**DOI:** 10.3390/molecules29010133

**Published:** 2023-12-25

**Authors:** Mehrnoosh Tavakoli, Bartłomiej Mazela, Wojciech Grześkowiak, Jędrzej Proch, Mirosław Mleczek, Waldemar Perdoch

**Affiliations:** 1 Faculty of Forestry and Wood Technology, Poznan University of Life Sciences, Wojska Polskiego 28, 60-637 Poznan, Poland; m.tavakoli@gau.ac.ir (M.T.); wojciech.grzeskowiak@up.poznan.pl (W.G.); miroslaw.mleczek@up.poznan.pl (M.M.); waldemar.perdoch@up.poznan.pl (W.P.); 2 Department of Pulp and Paper Technology, Gorgan University of Agricultural Sciences & Natural Resources, Gorgan 4913815739, Iran; 3 Faculty of Chemistry, Adam Mickiewicz University, 89B Umultowska Street, 61-614 Poznan, Poland; jedrzej.proch@amu.edu.pl

**Keywords:** fire performance, grafted cellulose, phosphorylation, mass loss calorimeter, mini fire tube, thermogravimetric analysis

## Abstract

Phosphorylated cellulose can be an intrinsic flame retardant and a promising alternative for halogenated fire inhibitors. In this study, the mixture of di-ammonium hydrogen phosphate (DAP) and urea (U), containing phosphate and nitrogen groups, was applied to attain fire inhibitor properties. Functional groups of cellulose were grafted with phosphorous by keeping the constant molar ratio of 1/1.2/4.9 between anhydroglucose units of cellulose/DAP/U in different concentrations of bleached kraft pulp. Phosphorus concentrations were determined using the ICP hrOES method, and paper sheets were made using the Rapid Köthen apparatus. The tensile strength of phosphorylated cellulose increased twice compared with unmodified cellulose when the phosphorous concentration increased to 10,000 g/kg. An increase in the tensile index comes from the higher freeness of pulp and cross-linking of the phosphorous group between cellulose fibers. Remarkable fire retardancy effects were achieved in cellulose concentrations above 5 wt%. The raised phosphorous concentration above 10,000 g/kg due to the phosphorylation process caused the formation of a char layer on a cellulose surface and the nonflammable gas emission. That effect was indirectly confirmed by reducing the combustion temperature and HRR by 50 and 45%, respectively. Due to increasing phosphorus concentration in cellulose sheets, cellulose’s fire and strength properties increased significantly.

## 1. Introduction

Contrary to appearances, flame-retardant paper and fire-resistant paper products, as a biodegradable material, have more and more applications and are a bold alternative to plastics and artificial fabrics. Non-flammable papers are often used as covering papers and applied under decorative wallpapers. Such papers may also constitute the outer layers of board materials (e.g., plasterboard). They are also used as core materials to create a honeycomb structure inside doors, as construction partitions, or to produce ceiling panels. Such papers should be light, but at the same time strong and often water-resistant. They are a popular choice in buildings where a high level of fire safety is required, such as high-rise buildings and public facilities. The prerequisite of using biodegradable and bio-based polymers, such as carbohydrates, proteins, phenolic compounds, and so on, is sufficient flame and heat resistance to be applied, e.g., for packaging purposes, military, and transportation industries. To achieve fire retardancy effects, incorporating different types of flame retardants into the biopolymers is common and effective. Recently, the tendency to use intumescent biodegradable polymers has been rising, as it is limited to the use of halogenated fire inhibitors due to environmental aspects, sustainable development, and prevention of the emission of toxic gases during combustion. Production of the so-called charring effect could lead to an improvement in biopolymer fire behavior. This means that the inclusion of phosphorus or nitrogen-containing flame retardants in polymer configuration after chemical modification, derived from the formed protective char layer, causes a reduction in the heat transferring to the sublayers, as well as the volatile fuels and heat release rate (HRR), eventually resulting in the appearance of the self-intumescent phenomenon [1,2,3].

Cellulose, as the most abundant carbohydrate on earth, either as a native form or its derivatives and nanocellulose, has great potential to interact with cationic or anionic groups due to its active functional groups. This interaction can potentially decrease its decomposition acceleration when exposed to flame. Cellulose degradation is usually started by the decomposition of aliphatic agents and a remarkable increase in furan and benzene rings at the condensed phases in the vicinity of the flame. According to literature sources, cellulose decomposition could be modified by employing flame retardants or heating conditions [4,5]. Phosphoric acid, ammonium hydrogen phosphate, di-ammonium hydrogen phosphate, ammonium polyphosphate, and phosphorus pentoxide are some examples of phosphate-containing intumescent flame retardants that are usually applied for cellulosic substance functionalization. The intumescence effect, which provides outstanding thermal insulation and is achieved using the mentioned materials, causes the formation of the foamed charred barrier layer on the surface of polymers, i.e., cellulose, to reduce the conduction of heat and mass from the heated substance [2,3,5,6,7,8]. During phosphate-based pretreatment, known as phosphorylation reactions, anionic phosphate groups as negative-charged agents lead to defibrillation of the cell wall of cellulose, which is considerably influenced by the molar ratio between anhydroglucose units of cellulose (AGU) and phosphorus-containing compounds, phosphorus degree of substitution, and also temperature and time of the phosphorylation pretreatment [2,9,10,11]. It is also proved that using urea and phosphorylated salts attains advantages such as enhancing the swelling of cellulose suspension during the reaction, better penetration of phosphoric compounds into cellulose structure, prevented cellulose decomposition, and better productivity of phosphorylated cellulose [2,4,11,12]. Also, some authors have emphasized that the presence of urea has a direct connection to reaching highly charged phosphate groups (>2 mmol/g) during the phosphorylation pretreatment of cellulose or nanocellulose, which are completely efficient for fire inhibitory effects [2,4,11].

It is important to investigate the retention of phosphate agents in the chemical structure of cellulose for optimizing phosphorylation reactions. Accordingly, Rol et al. [4] have declared that limited grafting is needed to better include the negative charges of phosphate elements on the cellulose proton positions and prevent fiber dissolution. They have also pointed out that phosphorylation productivity is tightly related to cellulose concentration (1 wt%, 2 wt%, and 5 wt%), the amount of urea, pH, and temperature. The viable proposed mechanism in the research of Belosinschi et al. [13] was based on applying reagents consisting of phosphoric acid and lauryl phosphate to attain the thermal stability of phosphorylated cellulose. This procedure was introduced as low-fiber degradation, low energy expenditure, and low use of chemical agents, with good efficacy of phosphorylated fibers. The effect was accomplished using (a) ammonia produced via urea degradation; (b) phosphoramidite formation as an intermediate agent; and (c) the phosphate–cellulose linkage (P-O-C) as three sequential reactions.

Apart from the fire-proofing behavior of phosphorylated cellulose, known as a more significant characteristic of phosphorylated fibers, the ion-exchange role of this modified cellulose is also noticeable [10,14,15,16,17,18,19]. Briefly, phosphorylated cellulose, as an ion exchanger, could be applied to remove heavy metals in effluent treatment [14,17,18], vitamin and protein chromatography, and for designing biomimetic substances [10,16,18].

Phosphorylated cellulose nanofibrils are introduced as a facile strategy to convert the macroscale cellulose to its nanoscale. For this purpose, cellulose isolated from wood or non-woody fibers is chemically functionalized via phosphate-containing salt and urea. Then, by using different mechanical treatments such as a high-pressure homogenizer [2], microfluidizer [5], and high-speed disintegrator [11], the cellulose chain is defragmented. For example, Messa et al. [11] recently mentioned that with an optimized molar ratio of 1/0.5/2 between anhydroglucose units of cellulose/di-ammonium hydrogen phosphate/urea, respectively, at pH 12, the anionic phosphate element was highly grafted to cellulose cationic hydroxyl groups extracted from sugarcane bagasse. Consequently, as a function of pH, gelation of highly phosphorylated nanocellulose was obtained. It is remarkable that in the case of employing high urea content in the phosphate-containing salt media for micro- or nanofibrillated cellulose production, problems arising from urea leaching should be considered for specific purposes like food or liquor packaging [5].

Phosphorylated cellulose, as an introduced and old procedure, has great potential to be applied as thermal insulation in biomedical fields, biochemical separation, or the textile industry; moreover, phosphorylated cellulose micro/nanofibrils have recently attracted great attention [4,8,12,18,20].

This study aimed to assess the influence of cellulose pulp concentration on the efficiency of phosphorylating cellulose and evaluate the fire properties of phosphorylated sheet papers. The scope of the work includes phosphorylation using a di-ammonium hydrogen phosphate/urea agent of different cellulose pulp concentrations (0.5 wt%,1 wt%, 2.5 wt%, 5 wt%, and 10 wt%) in an aqueous medium. The quantitative measurement of phosphorus retention according to the different cellulosic suspension concentrations was evaluated using the ICP hrEOS method. The pH value before and after the filtration and washing process was controlled. The fire behavior of phosphorylated cellulosic sheets was determined using two experiments: a mini fire tube and a mass loss calorimeter. Tensile properties of phosphorylated sheets and freeness of pulps that have direct relevance were evaluated.

## 2. Results and Discussion

### 2.1. Freeness, pH, and the Grammage of Different Phosphorylated Cellulose Pulps

The freeness of phosphorylated pulps is directly related to the initial concentration of cellulose suspensions (Table 1). Implementing phosphorylated groups to cellulose pulp in a low concentration reduces the pulp freeness. However, further increasing the concentration of pulp and simultaneously increasing the concentration of negatively charged phosphorus groups, which can be grafted to the carbon, promotes the water retention capacity of pulps. This observation is also described in the literature, namely that higher freeness could lead to higher defibrillation and flexibility of fibers, consequently enhancing the strength properties of papers. In other words, including hydrophilic phosphate groups in the cellulose chain acted as a refining or beating process and enhanced internal fibrillation between fibers. These processes are known from the literature, and the creation of P-O-C bonding on the cellulose surface was reported in the following reports [2,4,5,13,20,21]. However, the problem of leaching urea and cellulose degradation at a high phosphorylation degree cannot be ignored in the case of some particular applications [20,22]. The possible cross-linking between carbon from the cellulose and phosphorus elements during phosphorylation was reported [4]. As the pulp concentration increases, the pH of the pulp decreases. In addition, paper with a lower pH and higher freeness produced sheets with a lower grammage. The leaching process of pulp reduced the impact of acidic phosphorous groups on pH reduction. This observation suggests that not all phosphoric groups were effectively grafted to cellulose, and not all grafted water-soluble phosphoric components were easily leached out from the pulp. A pH reduction probably promotes the leaching of cationic components (e.g., Ca^2+^, and Na^+^) that come from cellulose production and finally reduces a paper sheet grammage. The leaching process of pulp reduced the impact of acidic phosphorous groups on pH reduction.

### 2.2. Phosphorus Content in Pulp

**Figure 1:**
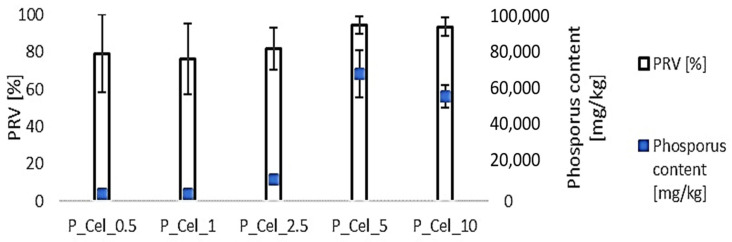
Phosphorus retention value and phosphorous content in a cellulose pulp.

The phosphorus concentration in pulp and the phosphorus retention value (%) are presented in Figure 1. It can be observed that the phosphorus content slightly increases with increasing pulp concentration from 0.5 to 2.5 wt%. However, the greatest influence on the content of phosphorus is not the concentration of the pulp but the method of its preparation, i.e., phosphorylated cellulose not thickened via filtration contained a several times higher concentration of phosphorus compared with filtered cellulose. For the process of cellulose phosphorylation, the curing stage seems to be crucial. Concentration via filtration, performed before curing, resulted in a significant phosphorus loss from the pulp. The probable reason for the higher concentration of phosphorous in cellulose phosphorylated with variant 5 wt% (P_Cel_5) than 10 wt% (P_Cel_10) was lower freeness of pulp, but this hypothesis should be confirmed in a future study. Irrespective of the phosphorus content in the cellulose material, its retention level was in the range of 70–90%.

### 2.3. Tensile Properties of Phosphorylated Cellulose Sheets

**Figure 2:**
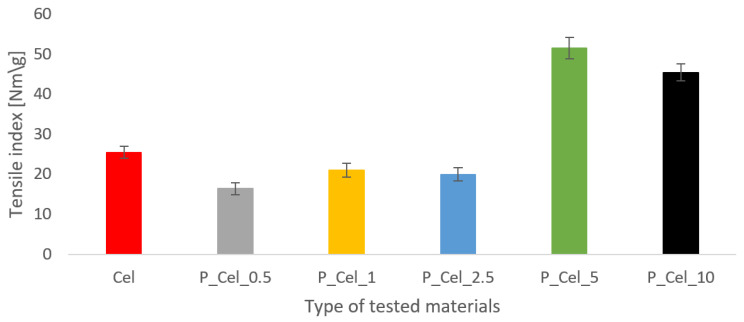
Tensile index (N*m/g) of paper sheets made with phosphorylated cellulose.

As shown in Figure 2, the phosphorylation process for P_Cel_5 and P_Cel_10 treatment processes significantly increased the strength properties of the paper. Those results can be easily correlated with phosphorus content results. A high concentration of phosphorous increased internal bonding in paper sheets due to the fact that phosphorous groups raised the freeness of pulp (e.g., [23]), and a curing process promoted a cross-linking effect of the phosphorylated cellulose. Consequently, the tensile strength for cellulose, including a high concentration of phosphorous, was increased almost two times for P_Cel_5 (45.9 N*m/g) and more than 1.5 times for P_Cel_10 (39.9 N*m/g) in comparison with the untreated sample (23.55 N*m/g). Due to the fact that the freeness of pulp consisting of phosphorous content in a low concentration was lower than untreated cellulose, the tensile index for treated samples was slightly lower than the control one. Indeed, having integrated mechanical properties is fundamental for phosphorylated cellulose sheets to act remarkably as fire inhibitors for various applications [4,6,7,24,25,26,27,28,29,30,31,32].

### 2.4. Fire Properties

#### 2.4.1. Mini Fire Tube Test

**Figure 3:**
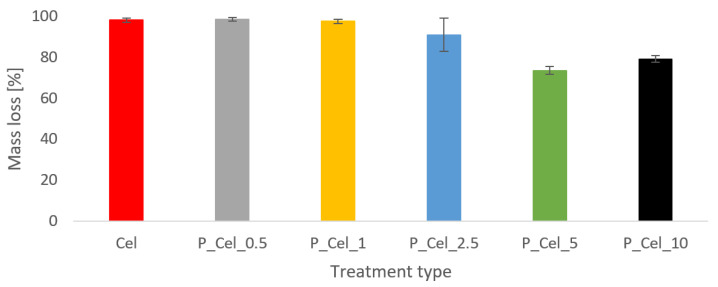
Mass loss of untreated and phosphorylated cellulose tested in an MFT method.

According to the mass loss percentage of samples obtained from MFT results (Figure 3), the untreated cellulose and samples consisting of approx. 4000 mg/kg of phosphorus were burned (mass loss approx. 99%). Increasing phosphorous concentration to approximately 11,000 g/kg slightly reduced an observed mass loss. In those cases, the standard deviation was quite high, suggesting that the phosphorous group’s distribution was not homogeneous. In detail, grafted oligophosphate moieties to cellulose structure in high concentrations (5 wt% and 10 wt%) reduced the mass loss of samples. The reason for this effect is likely connected with the creation of a char barrier layer and nonflammable gas emission during combustion, significantly retarding the endothermic depolymerization of glucopyranose units of cellulose [3,4,7,21,26,27,28,29]. It is worth noting that the maximum temperature of combustion (Table 2) was reduced even when the phosphorous concentration was low (above 4000 g/kg). This effect can be explained through the theory of nonflammable gas emission. This means the release of noncombustible volatile products derived from forming the protective barrier layer is significantly increased in modified samples [21,23,24,26,27,28,29,30]. As some authors declared [3,4,7], when phosphate salts provide a more acidic medium (lower pH), even after the filtration and leaching process, the self-extinguished behavior of phosphorylated samples is more prominent after removing the flame. A cellulose treatment does not impact the time to sample ignition and the time to reach a maximum combustion temperature.

#### 2.4.2. Crucial Results from the MLC Test

**Figure 4:**
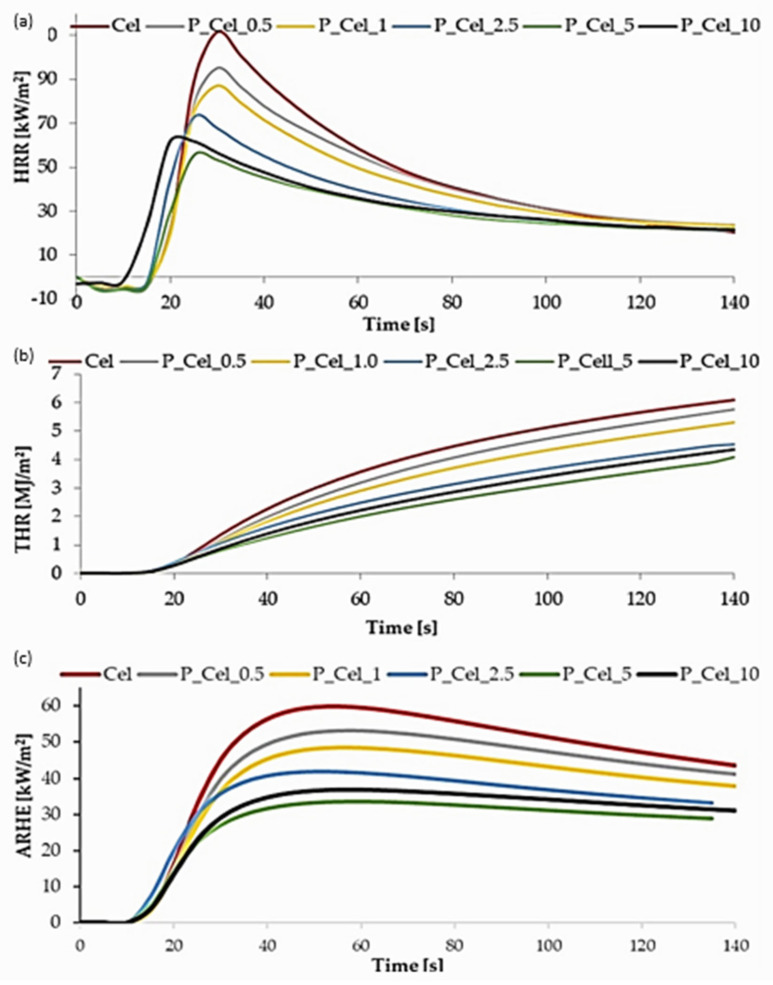
Cone calorimeter test of untreated and phosphorylated cellulose: (**a**) heat release rate; (**b**) total heat release; and (**c**) average rate of heat emission.

The fire-resistance properties of modified samples, particularly in high concentrations of cellulosic suspension, were considerably improved compared with the control sample exposed to 35 kW/m^2^ of irradiative heat flux during MLC tests (Figure 4a–c). Following an HRR curve, it was easy to observe that a fast-growing value at the beginning of the burning process was well-recognized and typical for thin materials [33]. Peak heat release rates (PHRR, kW/m^2^) and heat release rates (HRR, kW/m^2^) were significantly correlated with phosphorous concentration in the samples. This means that the PHRR and HRR were reduced when the concentration of phosphorous was raised (Figure 4a). An analysis of the PHRR value of cellulose samples consisting of more than 10,000 mg of phosphorous per 1 g of cellulose (P_Cel_5 and P_Cel_10) in comparison with unphosphorylated cellulose allowed an observed reduction in peak value of approx. 50%. Because their calculation is based on HRR results, total heat release and average heat emission rate correlate with the amount of grafted phosphorous in cellulose sheets (Figure 4b,c, respectively). The mass loss calorimeter test proved an observation of results from the MFT test. According to the literature, the formation of a carbon char layer and emission of nonflammable gasses during a fire action of phosphorylated sheets can swiftly generate self-extinguishing behavior, which appeared and probably prevented flame propagation during the test [33,34]. Moreover, PHRR, EHC, time to ignition, and time to flameout values are presented in Table 3. The average time to ignition of the phosphorylated samples was similar to the ignition time of the control samples. The recorded time to flameout was the longest in the case of the control trials and slightly reduced when the phosphorus values were below 4000 mg/g. Increasing phosphorous content above 10,000 mg/g reduced the time to flameout by approximately 30% in comparison with untreated cellulose.

#### 2.4.3. Crucial Results from the TG Test

**Figure 5:**
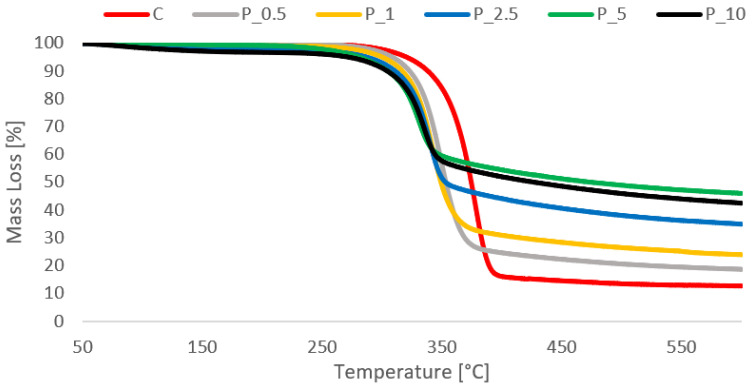
TG curves of phosphorylated material in the temperature range 40–600 °C.

Cellulosic material shows a single-stage pyrolysis reaction during heating in a nitrogen atmosphere (Figure 5). According to the literature data, the pyrolysis of cellulose (nitrogen conditions) is defined by two pathways involving the decomposition of the glycosyl units to char at a lower temperature and the depolymerization of such units to volatile products at higher temperatures [35,36]. Reactions of dehydration, desaturation, and glycosidic link breakage result in the levoglucosan formation. Levoglucosan readily decomposes into volatile compounds at temperatures lower than 400 °C [6,37]. The decomposition of control samples occurred between 290 °C and 404 °C. Phosphorylated samples also showed a single-stage pyrolysis reaction, with a shift in the onset of pyrolysis to lower temperatures, regardless of phosphorus concentration, compared with the control samples (Table 4) [38]. In comparison with the untreated cellulose, the presence of phosphorus in the samples caused earlier dehydration of cellulose toward char formation and a high reduction in the cellulose decomposition temperature [2]. As revealed by the mass loss value, the final residue at the end of the test was approximately 10% for untreated cellulose and 40% for phosphorylated samples with high phosphorous content.

## 3. Materials and Methods

A commercial bleached softwood kraft fiber (average length = 2100 μm, average width = 30.0 μm, coarseness = 135 μg/m, ash content = 0.25%, brightness = 89.5%, and pH = 4.8), as a dried form, was supplied for making sheets and the preparation of the cellulosic suspension. All the following chemicals were consumed as received: di-Ammonium hydrogen phosphate ((NH_4_)_2_HPO_4_, 99%, CAS no, 7783-28-0, ChemPur, Piekary Śląskie, Poland) and urea (CO(NH_2_)_2_, 99.5%, CAS no, 57-13-6, ChemPur, Poland). Deionized water was applied for all phosphorylation reactions.

### 3.1. Phosphorylation of Cellulosic Suspension

Phosphorylated cellulose production was described by Rol et al. [4]. Cellulosic pulps with 14 ± 0.8 Schopper–Riegler degrees (°SR) (ISO 5267 [39], Schopper–Riegler apparatus, Kontech, Poland) were mixed with the di-ammonium hydrogen phosphate and urea for 30 min. Cellulose pulp in five different concentrations (0.5 wt%,1 wt%, 2.5 wt%, 5 wt%, and 10 wt%) was mixed with the di-ammonium hydrogen phosphate and urea by maintaining the molar ratio of 1/1.2/4.9. Cellulose suspensions containing 0.5%, 1%, and 2.5% were filtered to 10%, but the 5 and 10% ones were directly dried at 105 °C in an oven. Then, the curing process was applied to the dried pulps at 150 °C for 1 h in an oven. Eventually, the dried pulps were diluted in water to reach 2 wt%, redispersed, and washed with boiling water (80 °C). For a better comparison between different concentrations of cellulosic suspensions, the pH of solutions before and after the filtration and washing process and the freeness of various pulp concentrations before making sheets (at least two evaluations of °SR according to ISO 5267 [39]) were measured.

### 3.2. Preparation of Phosphorylated Cellulose Sheets

Phosphorylated cellulose sheets (diameter = 200 ± 0.1 mm, thickness = 0.5 ± 0.09 mm) were produced using Rapid Köthen sheet former (Labor-Meks, Łódź, Poland). The following treatments were applied in this study: C (blank sample), P_Cel_0.5 (phosphorylated sample, 0.5 wt%), P_Cel_1 (phosphorylated sample, 1 wt%), P_Cel_2.5 (phosphorylated sample, 2.5 wt%), P_Cel_5 (phosphorylated sample, 5 wt%), and P_Cel_10 (phosphorylated sample, 10 wt%).

### 3.3. ICP hrOES Analysis of Phosphorus Concentration

The phosphorus content (mg/kg) was measured in pulp before and after leaching in different phosphorylated suspensions. The dry mass of pulp was accurately weighed (0.30 g (±0.01 g)) and then digested in 7 mL of nitric acid (65%; Sigma-Aldrich, St. Louis, MA, USA) in closed Teflon containers at 180 °C (20 min ramp time, 20 min hold time, and 20 min cooling down time) in the microwave digestion system Mars 6 Xpress (Mars 6 Xpress, CEM, Matthews, NC, USA). After digestion, samples were diluted with water (purified in the MilliQ water purification system (Merck Millipore, Darmstadt, Germany) to a total volume of 15.0 mL. The inductively coupled plasma high-resolution optical emission spectrometer ICP hrOES PlasmaQuant 9100 Elite (Analytik Jena, Jena, Germany) was used for phosphorus determination. The following conditions were used: radio frequency power 1.20 kW, plasma gas flow 12.0 L min^−1^, nebulizer gas flow 0.50 L min^−1^, auxiliary gas flow 0.5 L min^−1^, axial plasma view, and emission line P 213.618 nm. The signal was measured in 5 replicates for 1 s each. Yttrium was used as an internal standard (Y 371.030 nm). The detection limit (as 3–sigma criterion) was 0.55 mg/kg. For accuracy control, the standard addition method was used. The phosphorus retention value (PRV) for phosphorylated pulps (%) was evaluated according to Formula (1):

PRV = (P_1_ ∗ 100)/P_0_ [%] (1)

P_0_—Phosphate content in phosphorylated pulps before the washing process [mg/kg].
P_1_—Phosphate content in phosphorylated pulps after the washing process [mg/kg].

### 3.4. Tensile Strength

A tensile strength apparatus (Zwick Roell Z005, Germany) was used to evaluate elongation at the break of the phosphorylated sheets. Before flammability and tensile tests, the samples were conditioned at 20 ± 1 °C and 50% relative humidity for at least 24 h.

### 3.5. Fire-Proof Evaluation

#### 3.5.1. Mini Fire Tube (MFT)

The MFT method was adapted and modified according to ASTM E69 (2022) [40]. The mini fire tube was performed as the initial experiment and modified technique of vertical flammability [34,41]. The aluminum profile tube (length = 20 cm, diameter = 20 mm) was placed on the analytical balance. The flame (height of 1 cm) originated from a gas burner as the heating source placed on a trestle. The temperature of exhaust gases was measured via a k-type thermocouple with a temperature range of −50–1200 °C. At least 10 repetitions of each sample (length = 10 ± 1 mm, width = 100 ± 1.5 mm, and initial weight = 0.35 ± 0.05 g) were used for the MFT measurement.

#### 3.5.2. Mass Loss Calorimeter (MLC)

Crucial fire retardancy parameters such as peak heat release rate (PHRR), heat release rate (HRR), total heat released (THR), and mean value of effective heat of combustion (EHC), as a function of time, were evaluated using the mass loss calorimeter (MLC) test, (ISO 13927) [42]. Three sample repetitions (100 × 100 × 0.5 mm) were used for the MLC test (FTT—Fire Testing Technology—UK). The heat flux of the cone was 35 kW/m^2^.

#### 3.5.3. Thermogravimetric Analysis (TG)

Thermal analysis was performed using a thermal analyzer (Perkin Elmer Pyris 1 TGA—Thermogravimetric Analyzer—USA) designed for thermogravimetric (TG) measurements, which ensures obtaining independent signals recorded under the same measurement conditions: the heating rate, atmosphere, and pressure. This analysis method provided high efficiency and allowed obtaining comprehensive information on the thermal characteristics of the tested sample derivatives. The measurement was performed in a nitrogen atmosphere with a temperature range of 40–600 °C and a heating rate of 10 °C min^−1^. A protective gas was used for the tests, nitrogen with a 20 mL/min flow, and a purge gas nitrogen (50 mL/min) was used.

## 4. Conclusions

This study characterized manufactured cellulose grafted with (NH_4_)_2_HPO_4_/urea compounds. The influence of pulp concentration for grafting value and fire properties of phosphorylated cellulose was controlled. The concentration of pulp suspension significantly increased the freeness of pulp, a parameter that greatly influenced strength properties. The ICP hrOES analyses confirmed that pulp concentration and its preparation process critically influence phosphorylation effects. The cellulose pulp suspension containing 5 wt% of AGU/(NH_4_)_2_HPO_4_/CO(NH_2_)_2_ with a molar ratio of 1/1.2/4.9 and freeness around 32° accumulated phosphorous groups more effectively than pulp concentrations of 0.5, 1, 2.5, or 10 wt%. Due to increasing phosphorus concentration in cellulose sheets, cellulose’s fire and strength properties increased significantly. In a final effect of fire behavior, the cellulose with the highest concentration of phosphorous group promoted a char layer on a cellulose surface and emitted nonflammable gases, which consequently reduced the temperature of combustion and HRR energy and increased the fire properties of the paper sheets. 

## Figures and Tables

**Table 1:** Freeness and pH of cellulose pulp and the grammage of produced sheet paper.

Treatment Type	Freeness (°SR) Cellulose Pulp	pH of Pulp before Washing	pH of Pulp after Leaching	Grammage of Cellulose Sheets (g/m^2^)
Cel	14	-	-	308.4
P_Cel_0.5	10	6.82	7.73	301.9
P_Cel_1	8.5	6.81	7.53	297.7
P_Cel_2.5	16.5	6.71	7.02	296.8
P_Cel_5	38	5.62	6.11	297.4
P_Cel_10	64.5	5.79	6.20	292.9

**Table 2:** Maximum combustion temperature of untreated and phosphorylated cellulose tested in an MFT method.

Treatment Type	Maximum Temp. of Combustion (°C)	Time to Reach the Max. Combustion Temperature (s)
Cel	567.2 ± 24.2	15.0 ± 1.1
P_Cel_0.5	395.7 ± 45.7	17.0 ± 1.9
P_Cel_1	276.8 ± 49.5	16.8 ± 4.1
P_Cel_2.5	289.1 ± 42.1	15.0 ± 1.7
P_Cel_5	255.3 ± 31.7	15.0 ± 2.3
P_Cel_10	286.1 ± 20.5	17.0 ± 3.9

**Table 3:** Maximum values of flammability parameters of untreated and phosphorylated cellulose tested in an MLC method.

Parameters (Time to Maximum Value (s))	Treatment Type					
	Cel	P_Cel_0.5	P_Cel_1	P_Cel_2.5	P_Cel_5	P_Cel_10
Peak of Heat Release Rate (pHRR) (kW/m^2^)	111.5 (25 *)	87.6 (22 *)	76.9 (22 *)	40.3 (17 * 6.67)	43.90 (15)	61.6 (20 *)
Effective heat of combustion (EHC) (MJ/kg)	34.7 (28 *)	6.3 (21 *)	1.5 (21 *)	16.8 (16 *)	1.5 (15 *)	6.3 (20 *)
Time to ignition (s)	10.0	10	10	9	9	10
Time to flameout (s)	34	24	24	19	19	21

* Time to reach a maximum value.

**Table 4:** Thermogravimetric analysis of tested variants.

Treatment Type	Thermolysis Temp. Area (°C)	Max. Temp. of Decomposition Speed (°C)	Mass Loss (%)	
			In Thermolysis Area	Whole to 600 °C
Cel	289.9–404.3	340–396.8	82.67	87.4
P_Cel_0.5	261.2–397.2	314.5–379	74.6	81.42
P_Cel_1	273.83–390.7	311.2–379.8	66.35	76.15
P_Cel_2.5	254.2–375.5	318.3–353.8	41.8	57.61
P_Cel_5	249.2–374	317–340.17	41.29	54.11
P_Cel_10	262.5–373.83	320.2–362.83	50.27	65.17

## Data Availability

The data presented in this study are available upon request from the corresponding author.
